# PDA-PRGCN: identification of Piwi-interacting RNA-disease associations through subgraph projection and residual scaling-based feature augmentation

**DOI:** 10.1186/s12859-022-05073-3

**Published:** 2023-01-17

**Authors:** Ping Zhang, Weicheng Sun, Dengguo Wei, Guodong Li, Jinsheng Xu, Zhuhong You, Bowei Zhao, Li Li

**Affiliations:** 1 Hubei Key Laboratory of Agricultural Bioinformatics, College of Informatics, Huazhong Agricultural University, Wuhan, 430070, China; 2 Shenzhen Institute of Nutrition and Health, Huazhong Agricultural University, Shenzhen, 518000 China; 3 Shenzhen Branch, Guangdong Laboratory for Lingnan Modern Agriculture, Genome Analysis Laboratory of the Ministry of Agriculture, Agricultural Genomics Institute at Shenzhen, Chinese Academy of Agricultural Sciences, Shenzhen, 518000 China; 4 Hubei Hongshan Laboratory, Huazhong Agricultural University, Wuhan, People’s Republic of China; 5 School of Computer Science, Northwestern Polytechnical University, Xi’an, 710129 China; 6 The Xinjiang Technical Institute of Physics and Chemistry, Chinese Academy of Sciences, Urumqi, 830011 China

**Keywords:** Piwi-interacting RNA, Disease, Subgraph projection, Feature augmentation, Graph convolutional network, piRNA-disease associations

## Abstract

**Background:**

Emerging evidences show that Piwi-interacting RNAs (piRNAs) play a pivotal role in numerous complex human diseases. Identifying potential piRNA-disease associations (PDAs) is crucial for understanding disease pathogenesis at molecular level. Compared to the biological wet experiments, the computational methods provide a cost-effective strategy. However, few computational methods have been developed so far.

**Results:**

Here, we proposed an end-to-end model, referred to as PDA-PRGCN (PDA prediction using subgraph Projection and Residual scaling-based feature augmentation through Graph Convolutional Network). Specifically, starting with the known piRNA-disease associations represented as a graph, we applied subgraph projection to construct piRNA-piRNA and disease-disease subgraphs for the first time, followed by a residual scaling-based feature augmentation algorithm for node initial representation. Then, we adopted graph convolutional network (GCN) to learn and identify potential PDAs as a link prediction task on the constructed heterogeneous graph. Comprehensive experiments, including the performance comparison of individual components in PDA-PRGCN, indicated the significant improvement of integrating subgraph projection, node feature augmentation and dual-loss mechanism into GCN for PDA prediction. Compared with state-of-the-art approaches, PDA-PRGCN gave more accurate and robust predictions. Finally, the case studies further corroborated that PDA-PRGCN can reliably detect PDAs.

**Conclusion:**

PDA-PRGCN provides a powerful method for PDA prediction, which can also serve as a screening tool for studies of complex diseases.

**Supplementary Information:**

The online version contains supplementary material available at 10.1186/s12859-022-05073-3.

## Background

Piwi-interacting RNAs (piRNAs), a special kind of small non-coding RNA molecules with 26–31 nucleotides, are important regulatory factors in multiple biological processes through interacting with PIWI proteins [1]. Recently, a variety of evidences have confirmed that piRNAs play significant roles in transposon silencing and heterochromatin [2]. Meanwhile, irregular expression or modifications of piRNA are highly associated with complex diseases [3–5]. Owing to their critical role as a type of potential biomarker, exploring piRNA-disease associations (PDAs) is not only helpful for revealing the molecular mechanisms of diseases at noncoding RNA level, but also critical for further boosting the diagnosis, treatment, and prevention of human diseases. Conventional biological wet experiments for uncovering PDAs are often afflicted with high cost and time-consuming. Hence, it would be imperative to construct efficient and accurate models for identifying potential PDAs via computational methods.

Over the past few years, despite several databases involved in piRNAs such as piRBase [6], piRDisease [7], piRPheno [8], and MNDR [9] have been released, experimentally verified PDAs are far from comprehensiveness. To date, only several computational models have been put forward. Among them, iPiDA-sHN adopted convolutional neural network (CNN) to extract features and trained Support Vector Machine (SVM) to select negative samples to identify potential PDAs [10]. Afterward, iPiDi-PUL extracted key features and conducted dimension reduction by principal component analysis over feature vector based on positive unlabeled learning [11]. GAPDA treated each known piRNA-disease association pair as a node in their reconstructed graph and employed graph attention network to make representation learning [12]. SPRDA applied piRNA/disease similarity network to form a duplex network, then predicted PDAs as a matrix completion problem by structural perturbation algorithm [13].

Despite their successes, these models either generally regard known PDAs as feature data in Euclidean space (e.g., iPiDA-sHN and iPiDi-PUL), or reconstruct an abstract graph derived from original PDAs to simply transform link prediction into node classification problem (e.g., GAPDA and SPRDA). We argue that PDAs are naturally rich in structural features as a linked graph. Consequently, by implementing the raw PDA data as the intrinsic structure of a PDA graph, more accurate predictions are possible.

Beside GAPDA and SPRDA, with recent in-depth advances on graph theory and network science, various biomedical entity association prediction (EAP) approaches based on graph convolutional network (GCN) [14] have been proposed. In view of that, fitting the actual PDAs in GCN may be beneficial. Therefore, it is promising to adopt GCN to model PDA data as heterogeneous graph capable of making precise PDA prediction.

In this paper, we proposed a method, PDA-PRGCN, which incorporated three sequential strategies into GCN to detect potential PDAs. Specifically, we first constructed piRNA-piRNA and disease-disease subgraph separately by projecting the PDAs as links in the piRNA-disease graph. Then, to obtain high-quality initial representations, residual scaling-based node feature augmentation was designed to initialize the node feature to be propagated and aggregated in GCN layers. Finally, we introduced a dual-loss mechanism in an end-to-end GCN training process: accurately predicting relations by cross entropy-loss; adaptively constraining binary classification error between sensitivity and specificity by sensitivity–specificity loss. To evaluate the performance of PDA-PRGCN, extensive in silico experiments were performed. PDA-PRGCN achieved AUC of 0.9464, AUPR of 0.9190 on main dataset under fivefold cross-validation (5-CV), outperforming existing state-of-the-art methods. Case studies further confirmed the efficacy of PDA-PRGCN on PDA prediction.

## Results

### Experiment design

To evaluate the overall performance and validate individual components of our model, comprehensive experiments were designed. First, 5-CV was conducted. To explore the effect of link embedding, clustering of positive and negative links was visually checked. Then, prediction performances were compared among PDA-PRGCN and state-of-the-art methods. Subsequently, we presented the following comparisons: projection subgraph verses similarity subgraph; data augmentation similarity subgraph verses original similarity subgraph; residual scaling-based node feature augmentation verses original feature; balanced samples verse unbalanced samples in term of positive-to-negative ratio. The comparisons were made to evaluate the components of PDA-PRGCN and each of their influences on PDA identification.

Prediction performance of PDA-PRGCN was evaluated using the area under the receiver operating characteristic curve (AUC) and the area under precision–recall curve (AUPR). Relevant evaluation metrics include accuracy, precision, recall and F1 (their definitions can be found in Additional file 1: Note S1). It is important to note that AUPR is more suitable than AUC on evaluating model performance with unbalanced samples which are overwhelmed by negative samples, since it punishes false positives more stringently.

For PDA-PRGCN, the parameter epoch was set to 12,000 after optimization. The learning rate was set to 0.002 and the dropout rate was set to 0.2.

### Performance of PDA-PRGCN and comparison with state-of-the-art methods

We conducted 5-CV to compare model performance. Following the setup of previous methods, we took known PDAs as positive samples and randomly selected the same number of unlabeled PDAs as negative samples. For each fold, randomly divided subset containing positive and equal-size negative samples were held out as training data and the rest were used as test data.

**Fig. 1:**
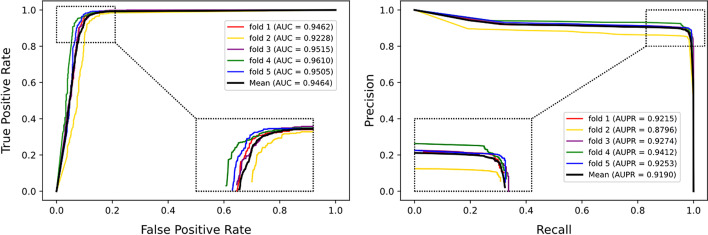
The ROC curves (left) and the (PR) curves (right) of PDA-PRGCN on main dataset under 5-CV

As shown in Table 1 and Fig. 1, PDA-PRGCN obtains mean AUC of 0.9464, as well as mean AUPR of 0.9190 on the main dataset, which validate the strategies of PDA-PRGCN on detecting potential PDAs. It is worth noting that although the AUPR in each fold is slightly lower than corresponding AUC, the average AUPRs keep at similar level with AUCs. This may be attributed to the weight put on the sensitivity–specificity loss function in the dual-loss optimization.

**Table 1:** Performance of PDA-PRGCN on main dataset under 5-CV

Fold	Accuracy	Precision	F1-score	Recall	AUC	AUPR
1	0.9043	0.9189	0.9027	0.8870	0.9462	0.9215
2	0.8942	0.8931	0.8944	0.8957	0.9228	0.8796
3	0.9123	0.9141	0.9121	0.9101	0.9515	0.9274
4	0.9188	0.9392	0.9169	0.8957	0.9610	0.9412
5	0.8921	0.9256	0.8877	0.8528	0.9505	0.9253
Mean	0.9044	0.9182	0.9028	0.8882	0.9464	0.9190

**Fig. 2:**
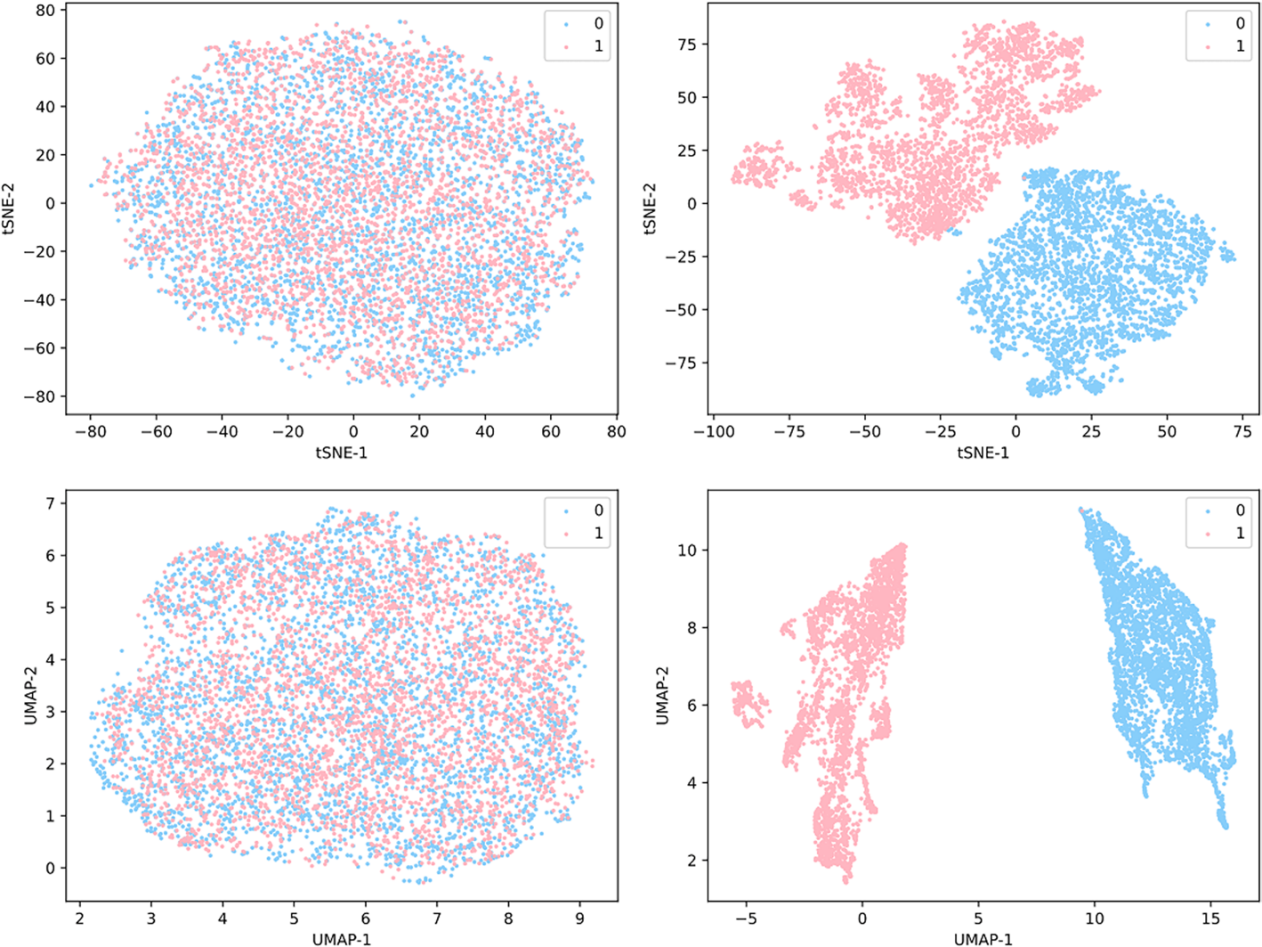
Visualization of link embedding before (left) and after (right) PDA-PRGCN via t-SNE (top) and UMAP (bottom) in 2D. Pink dots denote true (1) associations between piRNA and disease, while blue dots are the opposite (0)

Furthermore, to comprehend learning abilities of PDA-PRGCN, we mapped the link embedding derived from node embedding by our model into a 2D space by t-SNE [15] and UMAP [16], respectively. As shown in Fig. 2, the predicted PDAs by PDA-PRGCN are spatially clustered for positive and negative links. The visualization suggests that piRNA-piRNA/disease-disease subgraph construction via median subgraph projection and residual scaling-based node feature augmentation are successful for both piRNAs and diseases.

Then, we compared our model with existing baseline methods, i.e., iPiDi-PUL, iPiDA-sHN, GAPDA, SPRDA and LPI-deepGBDT [17]. For practical machine learning applications, it is an effective strategy to select negative samples. iPiDA-sHN and iPiDA-PUL do well in this point. The main aim of our study is to focus on the model performance and generalization ability on balanced sample structure using random negative samples that did not make any selection. To better assess the performance of PDA-PRGCN, we compared the deep learning-based model (i.e., LPI-deepGBDT) in which its feature space was designed in Euclidean space.

As shown in Table 2, our model has optimal prediction performance in six evaluation metrics, when applied on independent piRDisease dataset. In comparison with iPiDA-sHN and iPiDA-PUL, the performance of PDA-PRGCN is completely advantageous, even when unselected its negative samples. For LPI-deepGBDT, despite its success in lncRNA-protein interaction identification, the model regarded molecular association data as feature data in Euclidean space and adopted CNN to extract features. PDAs are naturally rich in structural features as a linked graph. GCN in our proposed PDA-PRGCN model gave better predictions as an encoder to model and represent features than the LPI-deepGBDT would do. Besides, although SPRDA was designed in Graph space and shows the next highest AUC, it does not get the same-level AUPR, which limits the performance to some extent. For the five methods with uneven AUPR, our model shows significant improvements. Together, the consistent prediction performances of PDA-PRGCN on the main and piRDisease datasets support the robustness of our model.

**Table 2:** Performance comparison of PDA-PRGCN against five baseline methods on piRDisease dataset

Method	Accuracy	Precision	F1-score	Recall	AUC	AUPR
LPI-deepGBDT	0.480	0.379	0.107	0.062	0.810	0.625
iPiDi-PUL	0.739	0.773	0.722	0.677	0.859	0.875
iPiDA-sHN	0.864	0.855	0.815	0.779	0.887	0.834
GAPDA	0.857	0.855	0.858	0.864	0.904	0.894
SPRDA	0.868	0.900	0.876	0.853	0.916	0.876
PDA-PRGCN	0.928	0.914	0.929	0.945	0.963	0.933

### Impact of various types of subgraph construction on model performance

Following the previous methods, that is, using similarity to construct subgraph, we explored the influence of two subgraph construction approaches: projection subgraph and similarity subgraph. Considering different node feature types, we first initialized node features for each node (piRNA or disease) respectively, then conducted the comparison to evaluate the impact of similarity-based subgraph construction under 5-CV. Besides, because we have already utilized the similarity to construct subgraph, we reset the feature for each node. Here, aside from constant, we can take role2vec as node pre-representation. Role2vec has proper representation effect and works well in unsupervised graph embedding [18]. Specifically, similar to model ablation study, we designed three similarity-based subgraph construction models below. For convenience, we use *OSG* to denote original similarity subgraph, *NF* to denote node feature and *rsSG* to denote residual scaling-based similarity subgraph.

- *OSG with constant NF*: it uses the original similarity to construct subgraph and set all node feature to 1.
- *OSG with role2vec NF*: it employs the original similarity to construct subgraph, but uses role2vec to learn node primary representation instead of constant.
- *rsSG with role2vec NF*: under the premise of using role2vec for NF, it adopts residual scaling-based similarity data augmentation to construct subgraph.

**Fig. 3:**
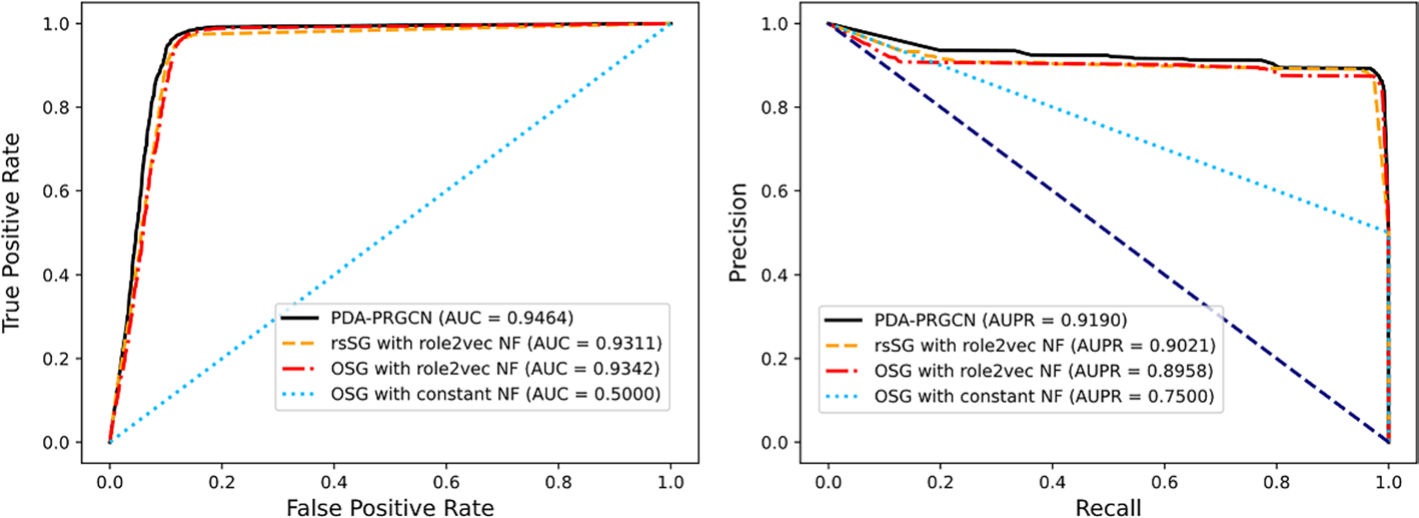
The ROC curves (left) and the PR curves (right) of four variations of subgraph construction

Figure 3 displays the performance comparisons between PDA-PRGCN and the three similarity-based subgraph construction methods in terms of AUC and AUPR. We observed that using original similarity to construct subgraph has poor performance. Particularly, for *OSG with constant NF*, it has AUC of 0.5 and AUPR of 0.75. For *OSG with role2vec NF*, considering the impact of node initial feature, after conducting node primary representation by role2vec, the model achieved an AUC > 0.9 and an AUPR > 0.8. Furthermore, to evaluate the effect of incorporating similarity data augmentation into model, we designed and tested *rsSG with role2vec NF*. As expected, its AUC gets the level of 0.93 and AUPR increases to 0.90. The improvement validates the strategy of incorporating data augmentation into similarity subgraph compared with original similarity subgraph. Although the setup of similarity subgraph with similarity data augmentation (i.e., *rsSG*) works well under role2vec, PDA-PRGCN remains the best solution.

### Impact of projection subgraph and node feature augmentation on model performance

In order to investigate the importance of projection subgraph (AM) and node feature augmentation (NIR), we designed two respective schemes. The first scheme was designed for NIR as following. For subgraph, we fixed the projection subgraph; for node feature, we only adopted original similarity profile with Stacked Autoencoder (i.e., SAE is used to reduce noise). To evaluate the impact of AM, the second scheme was designed as following. For node feature, we fixed node features augmentation; for subgraph, we integrated original similarity profile information into the projection subgraph. Here, the rationale of inputting original similarity profile information into the projection subgraph is to examine the influence of perturbations on projection subgraph in our model.

**Fig. 4:**
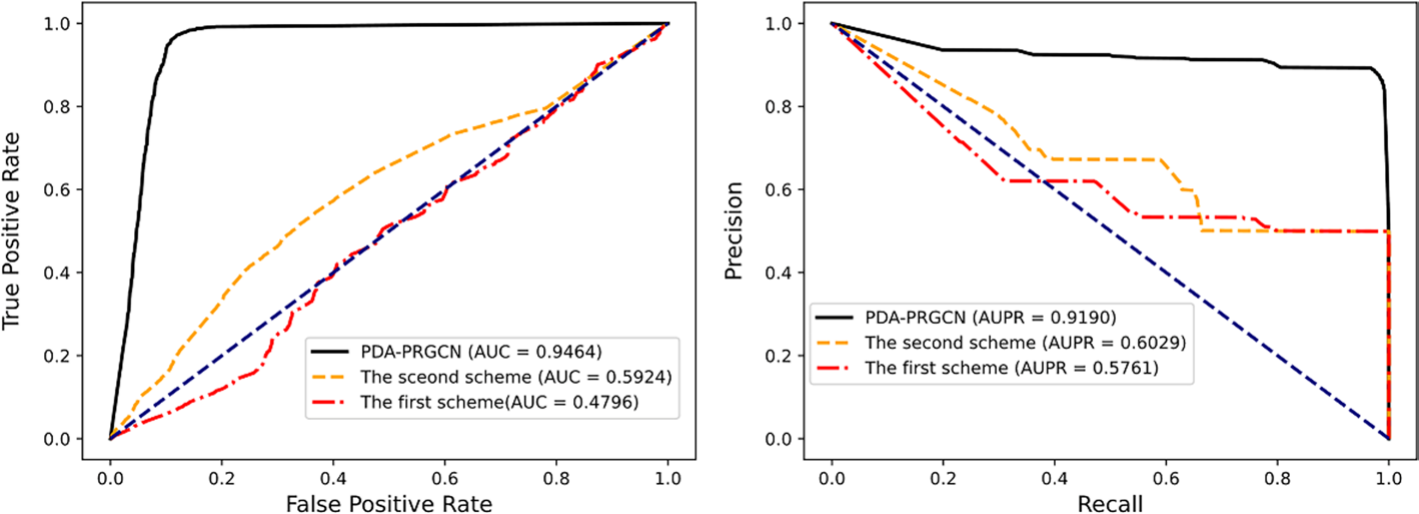
The ROC curves (left) and the PR curves (right) of three schemes

As shown in Fig. 4, without node feature augmentation, the first scheme performs poorly. By contrast, owing to the incorporation of the residual scaling-based node feature augmentation, the AUC and AUPR of PDA-PRGCN increase dramatically. It indicates node feature augmentation is a promising strategy for detecting potential PDAs. For the second scheme, with some perturbations on similarity profile information, the prediction performance decreases sharply. It suggests that the model is sensitive to similarity profiles even if node feature augmentation is not changed, thus substantiating the significant roles of projection subgraph in recognizing possible piRNA-disease links. Together, the experiments showed that for node feature augmentation and projection subgraph, removing either one will seriously hinder the performance of prediction. Thus, node feature augmentation and projection subgraph are complementary to each other in PDA-PRGCN. Integrating them both into our model can jointly enhance the PDA prediction.

### Impact of unbalanced sample structure on model performance

For practical applications, it is important to evaluate model performance on unbalanced sample structure in terms of positive and negative proportion. We built three sample setups with positive-to-negative ratio of 1:1, 1:5, and 1:10, respectively. Then we trained and tested our model on the samples under 5-CV. Since AUPR punishes false positives more severely than AUC, it is more instructive on model performance when negative samples are much more than positives. As shown in Table 3, PDA-PRGCN has reasonable performance in terms of AUPR in all the samples. As expected, AUPR decreases with the decease of positive-to-negative ratio from 1:1, 1:5 to 1:10. Nevertheless, the lowest AUPRs (0.5952 on main dataset, and 0.6263 on piRDisease dataset) keep at a moderate and practically acceptable level. This behavior suggests a weak dependency of model performance on sample structure. In total, our method performs quite well in samples with a wide range of positive-to-negative ratios.

**Table 3:** Performance of PDA-PRGCN for samples with different positive-to-negative ratios

Dataset	Ratio	Accuracy	Precision	F1-score	Recall	AUC	AUPR
Main dataset	1:1	0.9044	0.9182	0.9028	0.8882	0.9464	0.9190
	1:5	0.9249	0.7526	0.7841	0.8186	0.9503	0.7267
	1:10	0.9320	0.6122	0.6286	0.6888	0.9439	0.5952
piRDisease	1:1	0.9278	0.9144	0.9286	0.9445	0.9628	0.9328
	1:5	0.9295	0.7285	0.8130	0.9209	0.9630	0.7435
	1:10	0.9344	0.5903	0.7165	0.9123	0.9641	0.6263

### Case studies

Case studies on breast neoplasm, renal cell carcinoma, head and neck neoplasms and alzheimer disease were conducted to identify the potential piRNAs associated with each of the four diseases, respectively. For fairness of comparison, we applied PDA-PRGCN to independent dataset in which we ensured node information of collected piRNA-breast neoplasm/renal cell carcinoma/head and neck neoplasms/Alzheimer disease data were included in our training dataset (the main dataset) without edge information. The top ten predicted breast neoplasm-related piRNAs, top ten predicted renal cell carcinoma-related piRNAs, top ten predicted head and neck neoplasms-related piRNAs and top five predicted Alzheimer disease-related piRNAs were used to assess the applicability of PDA-PRGCN. As shown in Table 4, the predicted piRNAs are confirmed by the independent piRDisease database. Together, the case studies further substantiate the superior performance of PDA-PRGCN on PDA prediction.

**Table 4:** Validation of the top ten predicted breast neoplasm-related, renal cell carcinoma-related, head and neck neoplasms-related and top five alzheimer disease-related piRNAs by piRDisease

Rank	Breast neoplasm		Renal cell carcinoma	
	piRNA	piRDisease	piRNA	piRDisease
1	piR-hsa-2117	Yes	piR-hsa-26940	Yes
2	piR-hsa-11360	Yes	piR-hsa-26131	Yes
3	piR-hsa-26441	Yes	piR-hsa-13940	Yes
4	piR-hsa-23317	Yes	piR-hsa-25786	Yes
5	piR-hsa-1282	Yes	piR-hsa-2117	Yes
6	piR-hsa-952	Yes	piR-has-9010	Yes
7	piR-hsa-11361	Yes	piR-has-12719	Yes
8	piR-hsa-12487	Yes	piR-has-1282	Yes
9	piR-hsa-6496	Yes	piR-has-11362	Yes
10	piR-hsa-27616	Yes	piR-has-28478	Yes

Rank	Head and neck neoplasms		ALZHEIMER disease	
	piRNA	piRDisease	piRNA	piRDisease
1	piR-hsa-28394	Yes	piR-hsa-23210	Yes
2	piR-hsa-27493	Yes	piR-hsa-18287	Yes
3	piR-hsa-28187	Yes	piR-hsa-1077	Yes
4	piR-hsa-23992	Yes	piR-hsa-1849	Yes
5	piR-hsa-1823	Yes	piR-hsa-1823	Yes
6	piR-hsa-28395	Yes	–	–
7	piR-hsa-1282	Yes	–	–
8	piR-hsa-15399	No	–	–
9	piR-hsa-28190	No	–	–
10	piR-hsa-23655	No	–	–

## Discussion

Based on the performance evaluation and experiments conducted, the advantages of PDA-PRGCN are summarized as follows. First, it introduced a median subgraph projection approach for subgraph construction to capture the most likely links between piRNAs/diseases based on local centrality. This treatment is distinct from the commonly used similarity construction approaches. The outstanding performance suggests the potential of applying the strategy on other EAP problems. Second, a residual scaling-based node feature augmentation was designed and leveraged for a compact and high-quality initial node feature representation. Sequence-based k-mer similarity profile of piRNA and semantic similarity profile of disease contains redundant information. As a feature augmentation technique, residual scaling can effectively improve the final embedding. Third, a dual-loss mechanism was introduced, which can optimize the discrimination of binary samples especially for data containing unbalanced positive and negative samples.

Most methods of heterogeneous graph/network-based EAP via GCN modeling, by their very nature, mainly focus on how to construct the subgraph and initially represent the node [12, 19, 20]. It should be noted that similarity can be implemented in two alternative ways: construct subgraph as similarity graph; characterize the node feature as similarity profile. The experiment on our model showed the first way performed not well for PDA prediction. Instead, it is better to utilize it as a kind of similarity profile for node feature. This way, the enhanced similarity profile information among entities can be preserved and propagated with the layer-wise aggregation via GCN, thus, to ensure proximity between similar node embeddings and separability between dissimilar ones. Working with projection subgraph, the enhanced node features in our model can effectively represent the initially node states for following graph convolution process.

The limited number of known PDAs relative to all piRNA-disease pairs lead to the issue of unbalanced sample composition. Notwithstanding, various work train and test their models on balanced data [21–23], thereby limiting their applications in many practical scenarios. Prevailingly, researchers tend to subsample negative samples or equivalently decrease their weights in the optimization process [24]. This way, information in the negative samples might be under-represented. In contrast, for PDA-PRGCN, we increased the proportion of negative samples in the data as typically practical scenarios. The AUC increases with the inclusion of more negative samples, thereby demonstrating the efficacy of incorporating extra negative samples. We also observed that although AUPR decreased significantly at the same time, it is still at a competitive and practically acceptable level. It supports the power of dual-loss mechanism on unbalanced binary samples. Together, they moderate AUPRs on severely unbalanced samples suggest PDA-PRGCN as a promising solution on this challenging task.

Although this study incorporated subgraph projection to facilitate potential PDA prediction, the method involved projection node screening that relies on degree distribution of original dataset as a relational graph. In the future, we plan to adopt weighted subgraph projection to further improve subgraph construction. With more sophisticated biological information incorporated, the power of subgraph projection can be fully utilized to decipher biological associations more efficiently and accurately.

## Conclusions

In this paper, we proposed PDA-PRGCN, a computational method for potential PDA identification. Its prediction performance was evaluated by various comparative experiments extensively. Compared with the existing methods, PDA-PRGCN shows outstanding performance on PDA prediction. Moreover, competitive AUCs and AUPRs of PDA-PRGCN on highly unbalanced samples support its applicability as a screening tool in practice, where positive PDAs only represent a small proportion of all piRNA-disease pairs.

## Materials and methods

### Datasets and preprocessing

The manual-curated piRNA-disease datasets were collected from publicly available piRBase2.0 and MNDR v3.1 as the main dataset used in our model. Here, we only chose those experimentally verified PDA pairs. The sequence information of each piRNA can be obtained in the two databases. We filtered out those non-human PDA pairs from MNDR v3.1. Besides, it is worth mentioning that many nodes (i.e., piRNAs) with degree = 1 exist in the datasets. Considering the influence of degree on graph-based methods, as shown in Additional file 1: Figs. S1 and S2, we filtered out the nodes with degree = 1. For a graph containing N nodes, the degree distribution is defined as follows:

1 $$P_{k} \, = \frac{{N_{k} }}{N}$$

where *P*_*k*_ is the degree distribution and *N*_*k*_ denotes the number of nodes with degree = k, and *P*_*k*_ should meet $\sum\limits_{k = 0}^{\infty } {P_{k} } = 1$.

Finally, after performing the inclusion of identifier unification, de-redundancy and deletion of the irrelevant items from the two databases (i.e., piRBase2.0 and MNDR v3.1), we got the main dataset including 3446 pairs of known associations among 1478 piRNAs and 24 diseases.

In addition, for independent piRDisease dataset, generally viewed as benchmark dataset by the baseline methods, it consists of 4350 piRNAs, 21 diseases and 4993 known PDAs. We employed it to make comparisons with the five baseline methods.

Medical Subject Headings (MeSH) descriptor data of diseases were downloaded from https://meshb.nlm.nih.gov/.

### Method overview

**Fig. 5:**
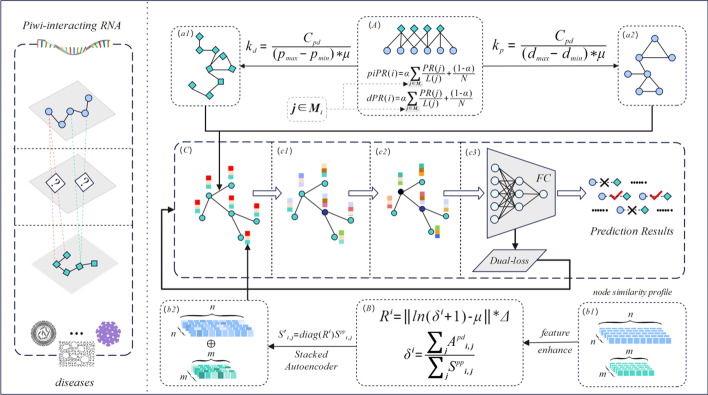
The flowchart of PDA-PRGCN. PDA-PRGCN contains the following three parts. First, a median subgraph projection algorithm (*A*) was designed to construct the disease-disease (*a1*) and piRNA-piRNA subgraph (*a2*). Secondly, a residual scaling-based feature augmentation algorithm (*B*) was applied for normalized similarity profile information (*b1*) to enhance the node feature (*b2*). Thirdly, we combined the above two parts using a GCN (*C*) with two layers (*c1*, *c2*) by dual-loss mechanism. Finally, a three-layer fully-connected neural network predictor (*c3*) was used to optimize model loss and output the probabilities of potential PDAs for PDA prediction

The backbone of a graph is node and edge. How to effectively mine structural information for edges and augment feature information for nodes side by side is a key point for graph-based methods. Based on this, the rationale of our method is to construct appropriate topology associations as adjacency matrix (AM) and enhance node features as node initial representation (NIR) on the heterogeneous graph of PDAs. This way, we can integrate the two components into a GCN and convert the problem of PDA prediction into a graph link prediction task. The flowchart is shown in Fig. 5.

### Composition of the PDA heterogeneous graph

Based on graph theory, we can treat the detection of potential PDAs as a link prediction task in graph. Thus, a heterogeneous graph consisting of the piRNA-piRNA subgraph, disease-disease subgraph and known piRNA-disease subgraph is established. Specifically, we can denote them by *A*^*pp*^, *A*^*dd*^, and *A*^*pd*^, then integrate the three subgraphs into a heterogeneous graph *G*. After aligning nodes of different subgraphs according to the node map, the final adjacency matrix *A* ∈ *R*^(*N*+*M*)× (*N*+*M*)^ of *G* is defined as follows:

2 $$A = \left[ {\begin{array}{*{20}l} {A^{pp} } & {A^{pd} } \\ {\left( {A^{pd} } \right)^{T} } & {A^{dd} } \\ \end{array} } \right]$$

where *N* is the number of piRNAs, and *M* is the number of diseases. *A*^*pp*^ denotes the projection matrix of piRNA-piRNA and *A*^*dd*^ denotes the corresponding disease-disease matrix, while *A*^*pd*^ denotes the known PDA matrix and (*A*^*pd*^)^*T*^ denotes its transposition.

Within the full graph, how to effectively construct *A*^*pp*^ and *A*^*dd*^ subgraph is a key point to infer potential PDAs. Different from those shallow embedding methods such as node2vec [25], LINE [26], and SDNE [27] etc., we more thoroughly considered node feature information combined with PDA heterogeneous graph to jointly learn the node representation in each convolution layer. The detailed construction procedure will be shown in the following sections.

### piRNA/disease subgraph construction

In this study, we adopted bipartite graph projection [28] to construct piRNA subgraph and disease subgraph individually. We assume that P = {P_1_, P_2_, …, P_n_} (n = 1478) is the set of piRNA nodes and D = {D_1_, D_2_, …, D_m_} (m = 24) is the set of disease nodes, while PDAs = {P_a_ D_b_} (1 ≤ a ≤ n and 1 ≤ b ≤ m) is the set of known piRNA-disease association. Given any P_p_ D_i_ ∈ PDAs and P_q_ D_i_ ∈ PDAs, (i.e., piRNA p and q are both related to disease i), we can infer the edge P_p_ P_q_ between P_p_ and P_q_ and construct the piRNA-piRNA subgraph. Same procedure applies to disease-disease subgraph construction. This way, the piRNA-piRNA and disease-disease subgraph were built using known PDAs.

It should be noted that piRNAs mostly point to only up to a few diseases. The default bipartite graph projection in the graph with a highly skewed degree distribution might void the assumption that piRNAs with similar functions are likely to be related to similar diseases. Specifically, for hub disease nodes in the piRNA-disease graph, a huge number, to the order of the square of disease degrees, of piRNA-piRNA connections will be generated using the projection rule. To control the inflation, we proposed a sampling procedure based on median node centrality. In general, for data with a long-tail distribution, the median and the region around it (e.g., the box in a boxplot) is appropriate to represent the data because the median is not sensitive to extremes. Instead of applying subgraph projection on all nodes, the projection is limited to certain number of nodes with centralities around the median. Therefore, we proposed a median centrality-based subsampling strategy as follows. For node centrality, we adopted the PageRank algorithm [29]. PageRank measures the importance of a node in relation to its linked nodes as following:

3 $${\text{PR}}(i) = \alpha \sum\limits_{j \in Mi} {\frac{{{\text{PR}}(j)}}{L(j)}} + \frac{(1 - \alpha )}{N}$$

where, *M*_*i*_ is the set of all webpages that have links to webpage *i*, *L*(*j*) is the number of links out of webpage *j*, *N* is the total number of webpages and *α* is set as 0.85 by default.

In the piRNA-piRNA/disease-disease subgraph, we selected *k* nodes with PR values around the median PR. The value of *k* can be decided as follows:

4 $$k = \frac{{C_{pd} }}{{\left( {d_{\max } - d_{\min } } \right) * \mu }}$$

where for piRNA projection subgraph, *C*_*pd*_ is the number of known PDAs. *d*_*max*_/*d*_*min*_ denotes the maximum/minimum degree of diseases, respectively. Similarly, for disease projection subgraph, corresponding *d*_*max*_/*d*_*min*_ denotes the maximum/minimum degree of piRNAs. *μ* is a hyperparameter as dilution factor chosen from {1.0,10.0,100.0, 1000.0, …}. Specifically, to limit *k* to an integer and in a reasonable range, *μ* was tested at various values and set to 1.0 for disease subgraph and 100.0 for piRNA subgraph, respectively. The procedure of constructing piRNA/disease subgraph is presented as the following pseudocodes:


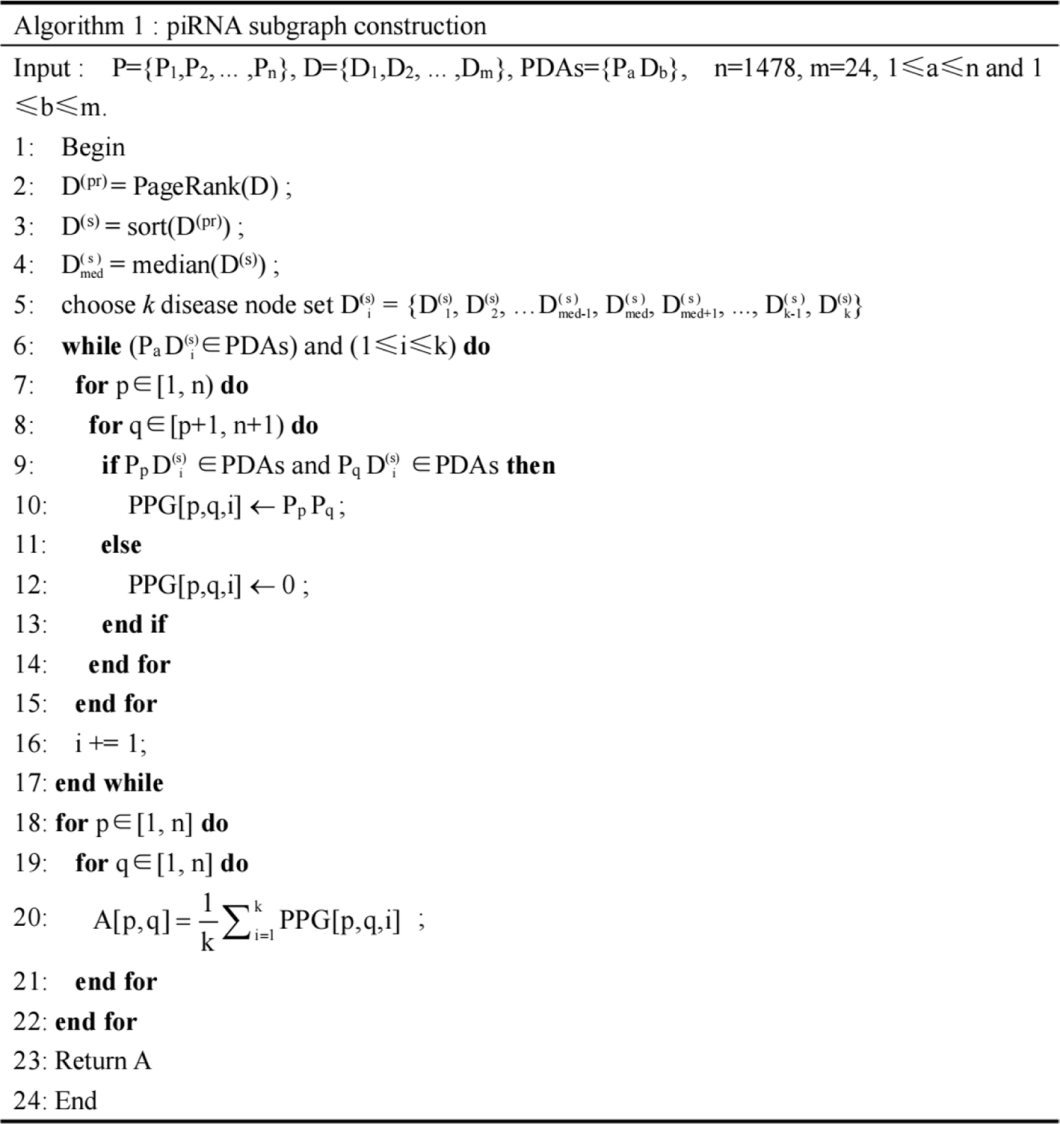


### Node feature construction

Similarity features between entities are crucial to characterize the node primary representation and are complementary with topological structure information. To felicitously apply them, we took them as a type of similarity profile information for node feature and proposed a residual scaling-based feature augmentation algorithm to comprehend and enhance node diversity.

### piRNA similarity profile

We adopted the Jaccard similarity coefficient [30] to calculate the k-mer similarity profile [31] for piRNAs. We first obtained the k-mer feature from original piRNA sequence information, where the k is empirically selected to 3(i.e.,3-mer). The Jaccard similarity coefficient between *x*_*i*_ and *x*_*j*_ was calculated as follows:

5 $${\text{S}}_{J} (i,j) = \frac{{|x_{i} \cap x_{j} |}}{{|x_{i} \cup x_{j} |}}$$

where *x*_*i*_ and *x*_*j*_ denote the binary feature vector of entity *i* and *j*. $|x_{i} \cap x_{j} |$ denotes the number of cases where both elements in *x*_*i*_ and *x*_*j*_ are equal to 1,and $|x_{i} \cup x_{j} |$ denotes the number of the cases where either the elements of *x*_*i*_ or *x*_*j*_ are equal to 1.

Considering the influence of sparsity in piRNA similarity profile matrix, the sparse entries can be estimated by mathematical expectation of the similarity between Jaccard similarity coefficient and Gaussian interaction profile (GIP) kernel similarity [32]. The GIP similarity between piRNA *i* and *j* was calculated as follows:

6 $${\text{S}}_{PG} (i,j) = \exp \left( { - n_{d} \left\| {A(i) - A(j)} \right\|^{2} } \right)$$

where *n*_*d*_ is a factor used to control the bandwidth of kernel. We can calculate *n*_*d*_ by normalizing the original kernel bandwidth $n^{{\prime }}_{d}$:

7 $$n_{d} = \frac{{n^{{\prime }}_{d} }}{{\frac{1}{k}\sum\limits_{i = 1}^{k} {\left\| {A(i)} \right\|^{2} } }}$$

where *k* denotes the number of all diseases and $n^{{\prime }}_{d}$ is usually set to 1. Thus, we can obtain the integrated piRNA similarity between *i* and *j* as follows:

8 $$S_{P} (i,j) = \left\{ {\begin{array}{*{20}l} {\frac{1}{2}{\text{S}}_{PG} (i,j)} & {{\text{if}}\;{\text{S}}_{p} {(}i,j{) = 0}} \\ {S_{J} (i,j)} & {{\text{otherwise}}} \\ \end{array} } \right.$$

### Disease similarity profile

In PDA-PRGCN, each disease including all related annotation terms obtained from MeSH descriptors can be represented by hierarchical directed acyclic graphs (DAGs). In general, a DAG can be expressed as *DAG* = (*T*(*d*), *E*(*d*)), for a given disease *d*. *T*(*d*) denotes *d* itself together with all its ancestor nodes, while *E*(*d*) denotes all relationships between nodes in the *DAG*(*d*). *D*_*d*_(*t*) of a disease *t* in a DAG to the semantics of disease *d* is defined as follows:

9 $$\left\{ {\begin{array}{*{20}l} {D_{d} (t) = 1} & {if\;t = d} \\ {D_{d} (t) = \max \left\{ {\Delta * D_{d} (t^{{\prime }} )\left| {t^{{\prime }} \in children\;of\;t} \right.} \right\}} & {if\;t \ne d} \\ \end{array} } \right.$$

where $\Delta$ is usually set to 0.5 according to previous studies. For a disease *d* to itself, the semantic contribution value is set 1, and with the distance between diseases increasing, the semantic contribution value will decrease. Thus, we can define the semantic value of disease *d* as following:

10 $${\text{DV}}(d) = \sum\nolimits_{t \in T(d)} {D_{d} (t)}$$

Following the method previously proposed [33], the semantic similarity score between disease *i* and *j* can be calculated by:

11 $$S_{D} (i,j) = \frac{{\sum\nolimits_{t \in T(i) \cap T(j)} {\left( {D_{i} (t) + D_{j} (t)} \right)} }}{{\sum\nolimits_{t \in T(i)} {D_{i} (t)} + \sum\nolimits_{t \in T(j)} {D_{j} (t)} }}$$

Similarly, with the same criteria as for piRNA, we can finally obtain the integrated disease similarity between *i* and *j* as follows:

12 $$S_{D} (i,j) = \left\{ {\begin{array}{*{20}l} {\frac{1}{2}{\text{S}}_{DG} (i,j)} & {{\text{if}}\;{\text{S}}_{D} (i,j) = 0} \\ {S_{D} (i,j)} & {{\text{otherwise}}} \\ \end{array} } \right.$$

### Residual scaling-based node feature augmentation

In general, individual residual profile has different contributions in similarity matrix. Being a kind of local-specific residual, it could efficiently represent the proportions of each row and thus characterize the importance of similarity profile for each node. Therefore, for each row in piRNA/disease similarity profile matrix, we have:

13 $$S^{{\prime }}_{i,j} = {\text{diag}} \left( {R^{i} } \right)S^{pp}_{i,j}$$

where, *S*^*pp*^ (*S*^*dd*^ for disease) is the matrix of original similarity profile of piRNA, diag denotes the diagonalization operator. *R*^*i*^ denotes the residual profile and can be defined as following.

14 $$R^{i} = \left\| {\ln \left( {\delta^{i} + 1} \right) - \mu } \right\|*\Delta$$

15 $$\delta^{i} = \frac{{\sum\nolimits_{j} {A^{pd}_{i,j} } }}{{\sum\nolimits_{j} {S^{pp}_{i,j} } }}$$

where, *A*^*pd*^ is the known PDA matrix. Here, we only considered the similarity of piRNA/disease distribution in *i*th row of *S*^*pp*^. *δ *^*i*^ is defined to describe the ratio of row degree distribution, while $\Delta$ ∈ {0.1,10,100} denotes the scaling factor and is used to shrink or enlarge the difference of distribution among different piRNAs/diseases. Evidently, the non-negative residuals are derived as *R*^*i*^ from *δ *^*i*^ and mean degree *µ*. Here, ln(·) and ||·|| denotes the Napierian logarithm operator and the non-negative absolute value operator, respectively. The procedure of residual scaling-based feature augmentation is presented as following pseudocodes (Here, the Stacked Autoencoder [34] was adopted to implement noise reduction.):


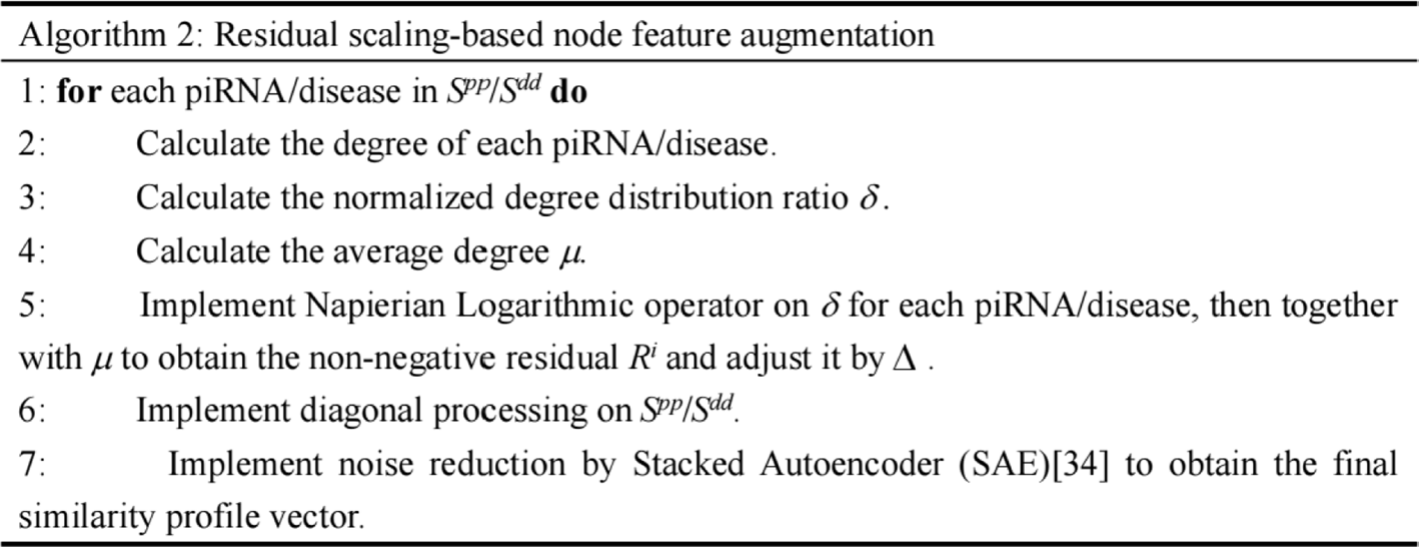


### Graph convolution network

We adopted GCN to learn final embedding and finished the link prediction task by an end-to-end mode. Specifically, we utilized the GCN with two convolution layers. The given graph adjacency matrix *A* and feature matrix *H* with the trainable weight vector *W* and the non-linear activation function *σ* jointly define the neural network *f* (⋅) as follows:

16 $$\mathop H\nolimits^{(l + 1)} = f\left( {\mathop H\nolimits^{(l)} ,A} \right) = \sigma \left( {\mathop {G\,H}\nolimits^{(l)} \mathop W\nolimits^{(l)} } \right)$$

where $\mathop {G = \, D}\nolimits^{( - 1/2)} \,A^{{\prime }} D^{( - 1/2)}$ with $A^{{\prime }} = A + I$ and *D* is the diagonal degree matrix of $A^{{\prime }}$, and ReLU is adopted as *σ*.

In view of the efficacy of PDA prediction, we designed a predictor of PDA-PRGCN by applying a three-layer fully-connected (FC) neural network to output the probability for potential links in the PDA graph, corresponding to feature extractor (i.e., GCN). Different from conventional dot product method [24, 35], our predictor is based on the end-to-end mode aiming for a better integration of embeddings and joint optimization of the proposed model as well as downstream tasks.

### Dual-loss mechanism

Together with the cross-entropy loss function applied to obtain the optimal classifications, the sensitivity–specificity loss function [36] was jointly adopted to train PDA-PRGCN. The dual loss is defined as follows.

17 $$Loss = L_{CE} + L_{SS}$$

18 $${\text{L}}_{{{\text{CE}}}} = - \sum\limits_{i = 1}^{N} {y^{(i)} \mathrm{log }\hat{y}^{(i)} + \left( {1 - y^{(i)} } \right)\log \left( {1 - \hat{y}^{(i)} } \right)}$$

19 $$L_{SS} = w * sp{ + (}1 - w{)} * se$$

where, $y^{(i)}$ is the true label and $\hat{y}^{(i)}$ is the predicted label. *w* = {0.1,0.01} represents sensitivity ratio, which can control the balance between sensitivity and specificity. Herein, *sp* denotes ${\text{Specificity}} = \frac{{{\text{TN}}}}{{{\text{TN}} + {\text{FP}}}}$ and *se* denotes ${\text{Sensitivity}} = \frac{{{\text{TP}}}}{{{\text{TP}} + {\text{FN}}}} \,$ with FN, TN, TP and FP denoting false negative, true negative, true positive and false positive, respectively.

## Supplementary Information

**Additional file 1.**  Degree distribution statistics of nodes in the main dataset and partial evaluation metrics definitions: **Figure S1.** Degree distribution of piRNAs in the main dataset; **Figure S2.** Degree distribution of diseases in main dataset; **Note S1.** Partial evaluation metrics definitions.

## Data Availability

All piRNA-disease association data in this study were downloaded from publicly available piRBase2.0 database, MNDR v3.1 database and piRDisease database, where the data provided by the three databases are open access and in accordance with the Declaration of Helsinki. Medical Subject Headings (MeSH) descriptor data of diseases were downloaded from https://meshb.nlm.nih.gov/. Source code of our models and training/testing datasets are available at: https://github.com/pzhangBIO/PDA-PRGCN

## Abbreviations

ROC: Receiver operating characteristic
PR: Precision–recall
AUC: Area under ROC curve
AUPR: Area under precision–recall curve
DAG: Directed acyclic graph
