# Supplementary material for: PDA-PRGCN: identification of Piwi-interacting RNA-disease associations through subgraph projection and residual scaling-based feature augmentation
Source: BMC Bioinformatics. 2023 Jan 17;24:18. doi: 10.1186/s12859-022-05073-3 (PMC9843905; doi:10.1186/s12859-022-05073-3)
Supplement: Supplementary file 1 — Additional file 1. Degree distribution statistics of nodes in the main dataset and partial evaluation metrics definitions: Figure S1. Degree distribution of piRNAs in the main dataset; Figure S2. Degree distribution of diseases in main dataset; Note S1. Partial evaluation metrics definitions. [file 12859_2022_5073_MOESM1_ESM.docx]

**PDA-PRGCN: identification of Piwi-interacting RNA-disease associations through subgraph projection and residual scaling-based feature augmentation**

**Ping Zhang^1^****^,2,3,†^, Weicheng Sun^1,2,3,†^, Dengguo Wei^1,2,3^, Guodong Li^1^, Jinsheng Xu^1^, Zhuhong You^5^, Bowei Zhao^6^ and Li Li^1,2,3,4,^**^*^

^*^Correspondence: [li.li@mail.hzau.edu.cn](mailto:li.li@mail.hzau.edu.cn) (LiL)

**^†^**Equal contribution.

^1^ Hubei Key Laboratory of Agricultural Bioinformatics, College of Informatics, Huazhong Agricultural University, Wuhan, 430070, China

^2^ Shenzhen Institute of Nutrition and Health, Huazhong Agricultural University, Shenzhen 518000, China

^3^ Shenzhen Branch, Guangdong Laboratory for Lingnan Modern Agriculture, Genome Analysis Laboratory of the Ministry of Agriculture, Agricultural Genomics Institute at Shenzhen, Chinese Academy of Agricultural Sciences, Shenzhen, 518000, China

^4^ Hubei Hongshan Laboratory, Huazhong Agricultural University, Wuhan, 430070, China

^5^ School of Computer Science, Northwestern Polytechnical University, Xi’an, 710129, China

^6^ The Xinjiang Technical Institute of Physics and Chemistry, Chinese Academy of Sciences, Urumqi 830011, China


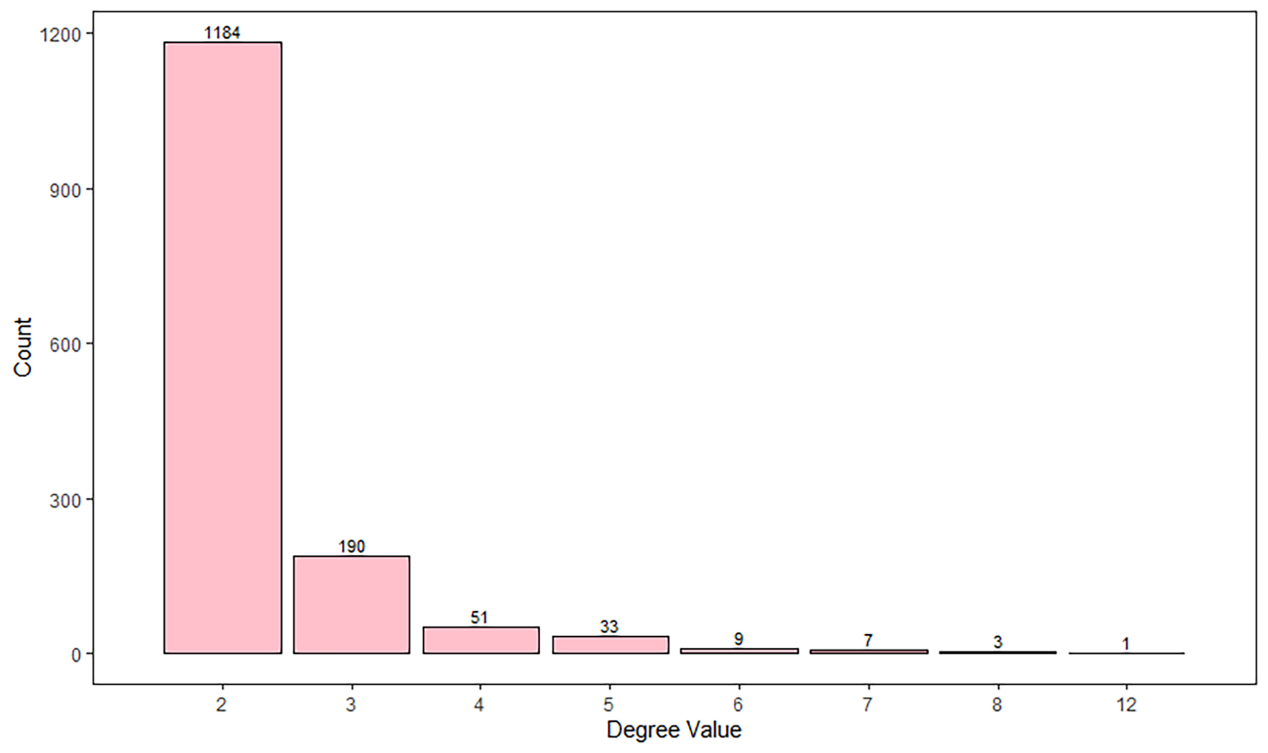


**Fig. S1**. Degree distribution of piRNAs in the main dataset.


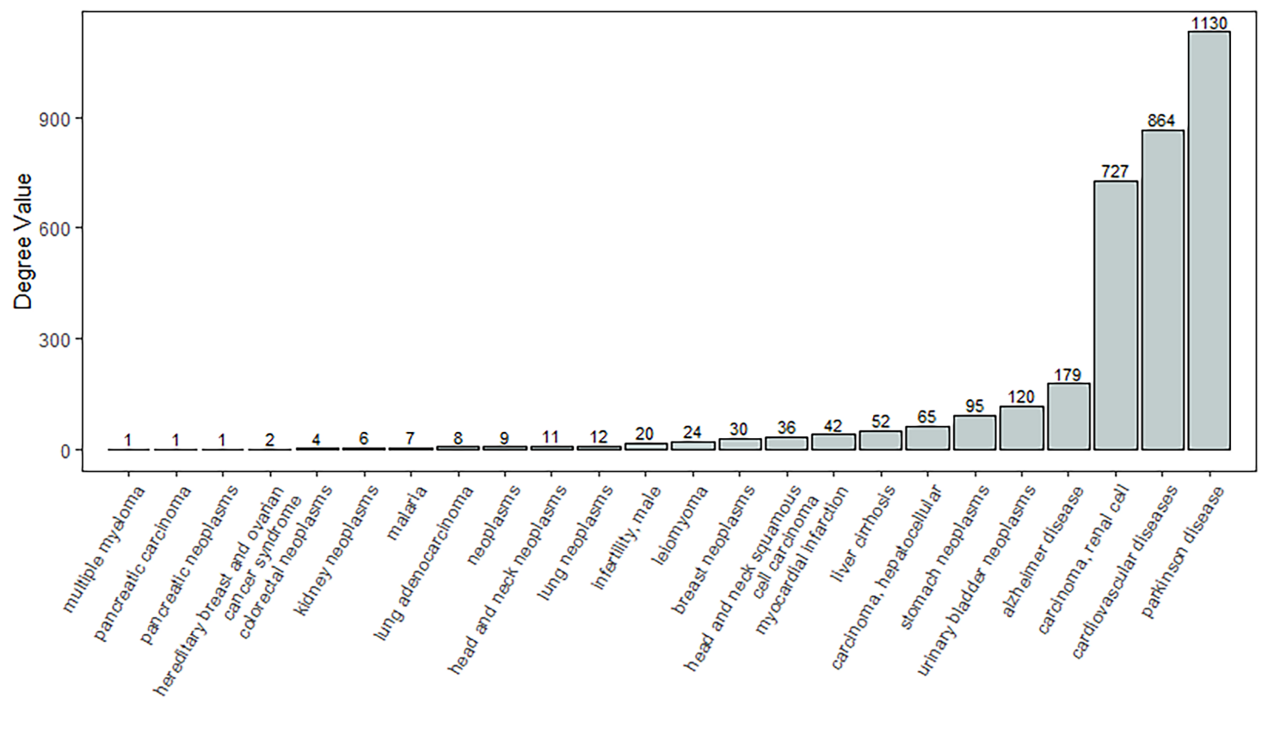


**Fig. S2**. Degree distribution of diseases in main dataset.

**Note S1 Partial evaluation metrics definitions**

$Accuracy=\frac{TP+TN}{TP+FP+TN+FN}$ （1）$\text{Precision}=\frac{TP}{TP+FP}$ （2）$\text{Recall}=\frac{TP}{TP+FN}$ （3）$\text{F1}=\frac{2\times\text{Precision}\times\text{Recall}}{\text{Precision}+\text{Recall}}$ （4）

where TP, FP, TN, and FN respectively represent the number of true positives, false positives, true negatives, and false negatives.
